# Beyond *Leishmania*: hidden trypanosomatid diversity reveals complex parasite-sand fly networks in southeastern Brazil

**DOI:** 10.1590/0074-02760250260

**Published:** 2026-05-01

**Authors:** Ana Paula Isnard, Amanda Caroline Corrêa Madureira, Gustavo Mayr de Lima Carvalho, Mariana Lourenço Freire, Daniel Moreira de Avelar, Lileia Gonçalves Diotaiuti, José Dilermando Andrade, Felipe Dutra-Rêgo

**Affiliations:** 1 Fundação Oswaldo Cruz-Fiocruz, Instituto René Rachou, Belo Horizonte, MG, Brasil

**Keywords:** Phlebotominae, Leishmania, Trypanosomatidae, phylogenetic analysis, vector-parasite interactions

## Abstract

**BACKGROUND:**

Sand flies (Diptera: Psychodidae) are well-known vectors of *Leishmania*, yet their associations with other trypanosomatids remain poorly understood. Expanding knowledge on these interactions is essential to elucidate the ecological diversity of parasites circulating in natural and periurban environments.

**OBJECTIVE:**

To characterise sand fly species composition and assess the diversity of trypanosomatids naturally infecting sand flies in the Serra do Cipó district, Minas Gerais, Brazil.

**METHODS:**

Sand flies were collected between 2023 and 2025 in the Mata da Tapera and nine surrounding peridomestic sites using Centre for Disease Control light trap (CDC-LT) and a Shannon trap. Females were examined by midgut dissection and screened individually for trypanosomatids using nested polymerase chain reaction (PCR) targeting the 18S rRNA V7-V8 region, followed by sequencing and phylogenetic analyses.

**FINDINGS:**

A total of 1,460 sand flies representing 21 species were collected, with *Pintomyia pessoai* (35.5%) being the most abundant. No flagellates were observed in 105 dissected females. Molecular screening of 730 females revealed 12 positives (overall positivity = 1.6%), including *Leishmania infantum* in *Pi. pessoai*, *Pi. christenseni*, and *Pa. barretoi*; *L. braziliensis* in *Pi. monticola*; and non-*Leishmania* taxa such as *Herpetomonas samuelpessoai*, *Novymonas esmeraldas*, a representative of Strigomonadinae, *Trypanosoma* sp. (Anura clade), and a lineage related to *Sergeia*.

**MAIN CONCLUSION:**

These findings confirm the circulation of *Leishmania* in Serra do Cipó while revealing hidden trypanosomatid diversity spanning at least five genera. The results suggest that sand flies may act as ecological “hubs”, transiently interacting with multiple parasite lineages beyond the classical *Leishmania* cycle, highlighting the need to broaden the ecological perspective in sand fly-parasite studies.

Sand flies (Diptera: Psychodidae: Phlebotominae) are highly diverse insects widely distributed in tropical and subtropical regions.^1^ Their medical relevance is primarily linked to their role as vectors of *Leishmania* (Kinetoplastea: Trypanosomatidae), the causative agents of leishmaniasis, a disease that remains a major public health challenge in Brazil and many other countries.^2^ Both cutaneous leishmaniasis (CL) and visceral leishmaniasis (VL) are endemic in Minas Gerais State, where periurban transmission has become increasingly frequent and has expanded into areas until recently considered free of the disease.^3^ ^,^ ^4^ ^,^ ^5^

Although the association between sand flies and *Leishmania* is well established, other trypanosomatids are increasingly reported in these insects, particularly in Brazil, including *Endotrypanum*,^6^ ^,^ ^7^ *Herpetomonas*,^8^ *Crithidia*,^8^ ^,^ ^9^ and *Trypanosoma*.^10^ These findings suggest that sand flies may interact with a broader range of monoxenous and dixenous parasites than traditionally recognised. While the epidemiological significance of these associations remains uncertain, their detection complicates the interpretation of molecular results in targeted surveillance of *Leishmania*, especially when using broad-range markers that can amplify multiple trypanosomatid lineages.

Periurban environments, shaped by habitat fragmentation, human settlement, and overlapping wild and domestic reservoirs, provide a unique scenario for exploring these interactions.^11^ In such contexts, shifts in sand fly composition and abundance may influence not only classical *Leishmania* transmission cycles but also the occurrence of other trypanosomatids.^12^ For *Leishmania*, it is well established that species naturally suited to exploit anthropised environments, such as *Lutzomyia longipalpis*, *Nyssomyia whitmani*, and *Migonemyia migonei*, play a central role in sustaining transmission in these areas.^13^ ^,^ ^14^ ^,^ ^15^ ^,^ ^16^ ^,^ ^17^ In contrast, whether sand flies that thrive in urban or periurban settings also contribute to the persistence or dispersal of other trypanosomatids, or merely reflect incidental associations, remains unclear. This uncertainty limits our understanding of parasite-vector interactions in anthropised landscapes.

Here, we address this gap by investigating sand flies from a forest fragment and peridomestic settings in Serra do Cipó, Minas Gerais, Brazil, to explore their species composition and to assess the occurrence of trypanosomatids. This region is part of one of Brazil’s major biodiversity hotspots and has previously reported the presence of sand fly vectors^18^ as well as autochthonous cases of canine visceral leishmaniasis (Municipal Health Department of Santana do Riacho).

## MATERIALS AND METHODS

*Study area and sand fly collection* - The study was conducted in the Serra do Cipó district (19º20′10.97″S, 43º37′48.75″W), municipality of Santana do Riacho, Minas Gerais, Brazil. This region forms part of the United Nations Educational, Scientific and Cultural Organisation (UNESCO)-designated Serra do Espinhaço Biosphere Reserve, a heterogeneous landscape that integrates Atlantic Forest and Cerrado elements and includes several conservation units, among them the Serra do Cipó National Park.

**Fig. 1:**
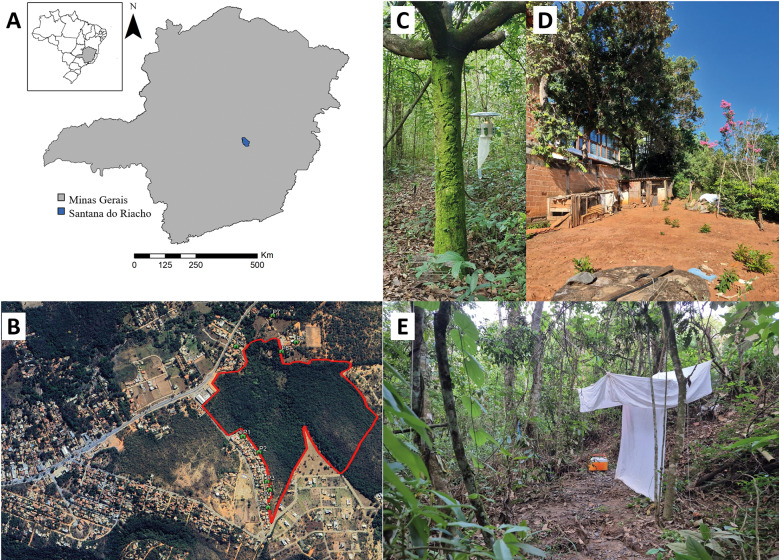
study area and sand fly sampling sites. (A) Location of Serra do Cipó district (Santana do Riacho, Minas Gerais, Brazil). (B) Mata da Tapera (MT) forest fragment (red polygon) and the nine peridomestic sites surveyed in this study. (C) Distribution of Centre for Disease Control light trap (CDC-LT) within the MT. (D) Peridomestic sampling sites surrounding the forest fragment where CDC-LT were deployed. (E) Shannon trap collection conducted in July 2024 at the MT.

Sand fly collections focused primarily on the Mata da Tapera forest fragment (MT) (19º19′50.84″S, 43º36′57.03″W) where five sampling campaigns were conducted between March 2023 and July 2024. Twenty Centre for Disease Control light trap (CDC-LT) were installed approximately 1 m above ground and operated from 6:00 p.m. to 6:00 a.m. for three consecutive nights in each campaign. In July 2024, a single Shannon trap collection was also performed from 6:00 p.m. to 9:00 p.m. In addition, nine peridomestic sites located in the immediate surroundings of the forest fragment were surveyed once as a preliminary assessment of sand fly fauna at the forest-household interface. Because only one campaign was conducted in peridomestic areas, these samples were not intended for direct ecological comparison with MT but rather to document species presence and explore potential parasite-vector interactions beyond the forest interior. The spatial distribution of all sampling sites is shown in Fig. 1.

Live sand flies collected in CDC-LT were removed from the cages using a Castro aspirator, whereas specimens from the Shannon trap were aspirated directly from the capture cloth. All live individuals were kept in transport boxes under controlled conditions [25ºC, 80% relative humidity (RH)] and were dissected for midgut examination. The remaining specimens were preserved in 70% ethanol for subsequent morphological identification and molecular screening. Dissected individuals were not included in the polymerase chain reaction (PCR) analyses.

For the peridomestic survey, nine sites were selected in January 2025 based on their location at the border of MT, as well as the presence of domestic animals and backyard vegetation. At each site, two CDC-LT were operated for three consecutive nights under the same conditions as described above. All sand flies from peridomestic collections were preserved in 70% ethanol.

*Natural infection of Trypanosomatidae* - Live females were transported to Fiocruz Minas (Belo Horizonte, Brazil) for midgut dissection on glass slides. Specimens were first chilled at -20ºC for 5 min and then washed three times in a 1:1 solution of distilled water and commercial detergent to remove bristles. After washing, the insects were maintained in sterile 1× phosphate-buffered saline (PBS) until dissection. The presence of promastigote forms in the gut was examined under light microscopy at 400× magnification, and the stage of the gonotrophic cycle was determined by analysing the ovaries and accessory glands.^19^ ^,^ ^20^

*Molecular detection of trypanosomatids* - Females not selected for midgut examination were nonetheless dissected individually for taxonomic identification. Each specimen was dissected with sterile needles on a slide containing 1× PBS, separating the head and the last three abdominal segments for morphological identification.^21^ The remaining body parts (thorax and abdomen) were stored separately in dry 1.5 mL tubes at -20ºC for subsequent molecular screening. Generic abbreviations follow the nomenclatural recommendations of Marcondes.^22^

DNA was extracted using the Gentra Puregene kit (Qiagen) following the manufacturer’s protocol. Extraction blanks (kit reagents without biological material) were included as negative controls. DNA purity and concentration were measured spectrophotometrically (NanoDrop One, Thermo Scientific), and the integrity of sand fly DNA was verified by PCR amplification of the *cytochrome c oxidase subunit I* (*COI*) gene using primers LCO1490 (5′-GGTCAACAAATCATAAAGATATTGG-3′) and HCO2198 (5′-TAAACTTCAGGGTGACCAAAAAATCA-3′) under previously described conditions.^23^ Reactions were performed in 25 μL containing 1× buffer, 2.5 mM MgCl₂, 0.2 mM dNTPs, 0.1 μM of each primer and 2 U Taq DNA. Cycling conditions were: 95ºC for 2 min; 35 cycles of 95ºC for 60 s, 54ºC for 60 s and 72ºC for 90 s; and a final extension at 72ºC for 10 min. For each PCR run, a no-template control (NTC) was included, consisting of the full reaction mix in which the sample DNA was replaced by nuclease-free water.

Detection of trypanosomatids was performed by nested PCR targeting the V7-V8 region of the *18S rRNA* gene, using primer pairs SSU561F (5′-TGGGATAACAAAGGAGCA-3′) / SSU561R (5′-CTGAGACTGTAACCTCAAAGC-3′) in the first reaction, and TRY927F (5′-CAGAAACGAAACACGGGAG-3′) / TRY927R (5′-CCTACTGGGCAGCTTGGA-3′) in the second.^24^ ^,^ ^25^ Reactions were performed in 25 μL containing 1× buffer, 2.0 mM MgCl₂, 0.2 mM dNTPs, 0.32 μM of each primer and 1 U Taq polymerase. First-round amplification used: 94ºC for 5 min; 30 cycles of 94ºC for 30 s, 55ºC for 60 s and 72ºC for 90 s; final extension at 72ºC for 5 min. The products of the first-round reaction using primers were diluted 1:25 in sterile water and 5 μL was used as template in the second round under the same conditions. Each reaction included *Leishmania braziliensis* DNA (MHOM/BH/1975/M2903) as a positive control and a NTC. Male sand flies were also processed as negative controls for both extraction and amplification.

PCR products were purified with the QIAquick PCR Purification Kit (Qiagen) and sequenced bidirectionally by the Sanger method. Forward and reverse chromatograms were inspected manually in FinchTV, and consensus sequences were assembled considering only bases with a minimum Phred quality score of 30. Ambiguous sites, when present, were evaluated by checking the chromatogram peak profiles and resolved by adopting the appropriate International Union of Pure and Applied Chemistry (IUPAC) ambiguity code when necessary. Primer sequences were trimmed from both ends prior to downstream analyses. The resulting consensus sequences were compared against GenBank using BLAST and subsequently used for phylogenetic inference.

*Phylogenetic analyses* - Sequences generated in this study were analysed together with reference sequences retrieved from the GenBank database. Reference sequences were selected by BLAST searches targeting the subfamilies Leishmaniinae, Phytomonadinae, Strigomonadinae and Trypanosomatinae, with *Bodo saltans* included as the outgroup. Sequences were aligned using the employing information from nocal structural interactions (E-INS-i) algorithm implemented in MAFFT v7.505.^26^ The V7-V8 alignment was subsequently refined by automated trimming with trimAl v1.5 using a gap-threshold of 0.5.^27^ Maximum likelihood (ML) inference was performed in IQ-TREE v2.4.0^28^ under the best-fit substitution model TIM3+F+I+G4 selected by ModelFinder.^29^ Node support was assessed with 1,000 ultrafast bootstrap replicates and 1,000 SH-aLRT tests.^30^ Bayesian inference was performed in MrBayes v3.2.7 under the GTR+F+I+G model for 5,000,000 generations, sampling every 100, with remaining parameters set to default.^31^

*Ecological analysis and interaction matrix* - To evaluate sampling completeness and estimate species richness, rarefaction and extrapolation analyses using the iNterpolation and EXTrapolation (iNEXT) framework were applied.^32^ ^,^ ^33^ Analyses were based on abundance data, considering Hill numbers for q = 0 (species richness), q = 1 (Shannon diversity, exponential form), and q = 2 (Simpson diversity). Sample-size and coverage-based curves were generated with 2,000 bootstrap replications to estimate 95% confidence intervals (CI). Chao 1 richness was also computed (EstimateS v9.1.0) for comparison with observed values.

To visualise vector-parasite associations, the Sankey diagram was generated in Flourish (https://flourish.studio) using the ‘arbitrary flows’ mode with automatic node ordering (‘Reduce overlaps’) to minimise line crossings.

*Ethical statements* - Sand fly collections were approved by the Authorisation and Information on Biodiversity System (SISBIO; permit number 86644-1). The study was registered in the National System for the Management of Genetic Heritage and Associated Traditional Knowledge (SisGen) under registration number A47F256. Collection performed at peridomestic settings were approved by the owners (anonymised here) sampling site locations are summarised in the Supplementary data (Table I).

## RESULTS

A total of 1,460 sand flies were collected during the study, with 1,226 from the MT (84%) and 234 from peridomestic areas (16%), representing 21 species across nine genera. *Pi. pessoai* was the most abundant species (35.5%), followed by *Pi. monticola* (23.6%) and *Ev. evandroi* (7.8%) (Table I). No engorged females were obtained in any of the sampling campaigns, which prevented blood-meal source analyses.

**TABLE I:** Sand flies collected with Centre for Disease Control light trap (CDC-LT) and a Shannon trap between 2023 and 2025 in the Mata da Tapera (MT) and in peridomestic settings surrounding MT in the Serra do Cipó district, Minas Gerais, Brazil

Sand fly	MT		Peridomestic settings		Total (%)
	♂	♀	♂	♀	
*Brumptomyia brumpti*	6	5	1	1	13 (0.9)
*Evandromyia bacula*	0	3	0	0	3 (0.2)
*Evandromyia cortelezzii*	11	17	23	18	69 (4.7)
*Evandromyia evandroi*	20	23	39	32	114 (7.8)
*Evandromyia teratodes*	0	2	0	0	2 (0.1)
*Evandromyia termitophila*	1	1	0	0	2 (0.1)
*Lutzomyia ischnacantha*	1	7	0	0	8 (0.5)
*Lutzomyia longipalpis*	26	4	54	5	89 (6.1)
*Micropygomyia longipennis*	2	4	0	0	6 (0.4)
*Micropygomyia quinquefer*	0	5	0	0	5 (0.3)
*Migonemyia migonei*	2	0	3	1	6 (0.4)
*Nyssomyia whitmani*	28	22	8	6	64 (4.4)
*Pintomyia christenseni*	29	52	0	7	88 (6.0)
*Pintomyia monticola*	88	250	4	2	344 (23.6)
*Pintomyia pessoai*	294	200	19	5	482 (35.5)
*Psathyromyia aragaoi*	46	22	0	0	68 (4.7)
*Psathyromyia barretoi*	0	23	0	0	23 (1.6)
*Psathyromyia brasiliensis*	0	3	0	0	3 (0.2)
*Psathyromyia lutziana*	3	6	0	0	9 (0.6)
*Sciopemyia birali*	0	1	0	1	2 (0.1)
*Sciopemyia sordellii*	6	13	0	5	24 (1.6)
Total (%)	563 (46)	663 (54)	151 (64.5)	83 (35.5)	1.460 (100)
	1.226 (84)		234 (16)		

Among the specimens collected in MT, 663 were females (54%) and 563 were males (46%). *Pi. pessoai* was the predominant species (N = 494; 40.2%), followed by *Pi. monticola* (N = 338; 27.5%) and *Pi. christenseni* (N = 81; 6.6%) [Supplementary data (Table II)]. In the Shannon collection conducted in July 2024, 52 sand flies were captured: 36 males, all identified as *Pi. pessoai*, and 16 females, comprising *Pi. pessoai* (N = 8; 50%), *Pi. monticola* (N = 4; 25%), *Mi. quinquefer* (N = 2; 12.5%), *Ny. whitmani* (N = 1; 6.3%), and *Pa. aragaoi* (N = 1; 6.3%).

**Fig. 2:**
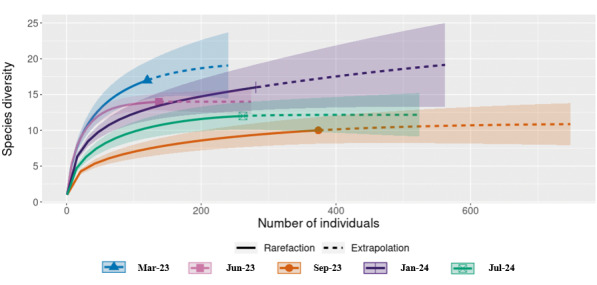
rarefaction and extrapolation of sand fly species richness in the Mata da Tapera (MT). Rarefaction (solid lines) and extrapolation (dashed lines) curves were generated using the iNterpolation and EXTrapolation (iNEXT) framework based on abundance data, with 2,000 bootstrap replications to estimate 95% confidence intervals (CI) (shaded areas). Species richness stabilised after the fourth collection campaign, with little increase predicted under extrapolation. Observed richness (21 species) coincided with the *Chao 1* estimator [21; standard deviation (SD) = 0.12], indicating that sampling effort was sufficient to capture local diversity.

The species accumulation curve stabilised from the fourth CDC collection campaign onward, indicating that sampling was sufficient to capture local richness (21 species). This was corroborated by the *Chao 1* estimator [*Chao 1* = 21; standard deviation (SD) = 0.12]. Rarefaction and extrapolation curves further indicated that additional sampling would contribute little to the observed diversity, given that the 95% CI of the extrapolated richness overlapped with the observed estimate (Fig. 2). Coverage-based rarefaction and extrapolation curves also indicated high sample completeness (> 95%) [Supplementary data (Figs 1-2)].

Natural infection was assessed by dissecting 105 live females, including 89 collected with CDC-LT and 16 from the Shannon trap (Table II). The most frequently dissected species was *Pi. pessoai* (N = 52; 49.5%), followed by *Pi. monticola* (N = 22; 21%) and *Ev. evandroi* (N = 8; 7.6%). Among the dissected specimens, 34% (N = 36) were classified as nulliparous and 66% (N = 69) as parous. No flagellates were observed in the examined samples.

**TABLE II:** Female sand flies collected in the Mata da Tapera (MT), Serra do Cipó district, Minas Gerais, Brazil, and tested for natural *Leishmania* infection

Sand fly	Mar 23	Jun 23	Set 23	Jan 24	Jul 24* (CDC/SH)	Total (%)
*Brumptomyia* sp.	-	3	-	-	-	3 (2.9)
*Evandromyia cortelezzii*	3	-	-	1	-	4 (3.8)
*Ev. evandroi*	1	-	6	1	-	8 (7.6)
*Lutzomyia longipalpis*	3	-	-	-	-	3 (2.9)
*Micropygomyia quinquefer*	1	-	2	-	0 / 2	5 (4.8)
*Nyssomyia whitmani*	-	-	-	-	0 / 1	1 (1.0)
*Pintomyia christenseni*	1	-	4	-	-	5 (4.8)
*Pi. monticola*	2	-	-	16	0 / 4	22 (21.0)
*Pi. pessoai*	4	-	13	14	13 / 8	52 (49.5)
*Psathyromyia aragaoi*	1	-	-	-	0 / 1	2 (1.9)
Total	16	3	25	32	13 / 16	105 (100)

*In July 2024, live females collected with both Centre for Disease Control light trap (CDC-LT) and a Shannon trap were used for the natural infection survey.

A total of 647 females collected in MT with CDC-LT were screened for trypanosomatids using nested PCR targeting the V7-V8 region of the *18S rRNA* gene. Ten specimens tested positive: *Pi. monticola* (N = 5), *Pi. pessoai* (N = 2), and one each of *Mi. longipennis*, *Pi. christenseni*, and *Pa. barretoi*. Sanger sequencing (Table III) and phylogenetic analysis of the V7-V8 region revealed the presence of *Leishmania infantum* in *Pi. christenseni* (N = 1), *Pi. pessoai* (N = 1), and *Pa. barretoi* (N = 1); *L. braziliensis* in *Pi. monticola* (N = 2); *Herpetomonas samuelpessoai* in *Pi. monticola* and *Pi. pessoai* (N = 1, each); *Trypanosoma* sp. in *Mi. longipennis*; *Novymonas esmeraldas* in *Pi. monticola* (N = 1); and a representative species within Strigomonadinae in *Pi. Monticola* (N = 1). All sequences have been deposited in GenBank under accession numbers PX260227-PX260238. The overall positivity rate for trypanosomatids in MT was 1.5%.

**TABLE III:** Trypanosomatids in polymerase chain reaction (PCR)-positive female sand flies (Nested PCR targeting the V7-V8 region of the *18S rRNA* gene) collected in the Mata da Tapera (MT) and in peridomestic settings of the Serra do Cipó district, Minas Gerais, Brazil

Access number (GenBank)	Sand fly	Collection date / site	Trypanosomatidae	Query cover	Identity
PX260227	*Pintomyia monticola*	Jan-24 / sylvatic	*Leishmania braziliensis*	100%	100%
PX260228	*Pi. monticola*	Jan-24 / sylvatic	*Leishmania braziliensis*	100%	100%
PX260229	*Pi. christenseni*	Jun-23 / sylvatic	*Leishmania infantum*	100%	100%
PX260230	*Psathyromyia barretoi*	Jun-23 / sylvatic	*Leishmania infantum*	100%	100%
PX260231	*Pi. pessoai*	Jul-24 / sylvatic	*Leishmania infantum*	100%	100%
PX260232	*Pi. monticola*	Jul-24 / sylvatic	*Novymonas esmeraldas*	100%	100%
PX260233	*Pi. monticola*	Jan-24 / sylvatic	*Herpetomonas samuelpessoai*	100%	100%
PX260234	*Pi. pessoai*	Jan-24 / sylvatic	*Herpetomonas samuelpessoai*	100%	100%
PX260235	*Pi. monticola*	Jan-24 / sylvatic	Trypanosomatidae sp.	100%	99.8%
PX260236	*Micropygomyia longipennis*	Mar-23 / sylvatic	*Trypanosoma* sp.	100%	100%
PX260237	*Nyssomyia whitmani*	Jan-25 / peridomestic	Trypanosomatidae sp.	100%	99.8%
PX260238	*Sciopemyia sordellii*	Jan-25 / peridomestic	*Trypanosoma* sp.	100%	100%

In peridomestic settings, a total of 234 sand flies were collected in January 2025 (males = 151; 64.5%; females = 83; 35.5%). This peridomestic survey represents an initial assessment of the forest-household interface. The most abundant species were *Ev. evandroi* (N = 71; 30.3%), *Lu. longipalpis* (N = 59; 25.2%), and *Ev. cortelezzii* (N = 41; 17.5%) [Supplementary data (Table III)]. All females were screened for trypanosomatids using nested PCR targeting the V7-V8 region of the *18S rRNA* gene, and two specimens tested positive: one *Sc. sordellii* collected at site P4 was positive for *Trypanosoma* sp., and one *Ny. whitmani* collected at site P5 was positive for a trypanosomatid related to the genus *Sergeia* (typically associated with biting midges). The overall positivity rate for trypanosomatids in peridomestic settings was 2.4%.

**Fig. 3:**
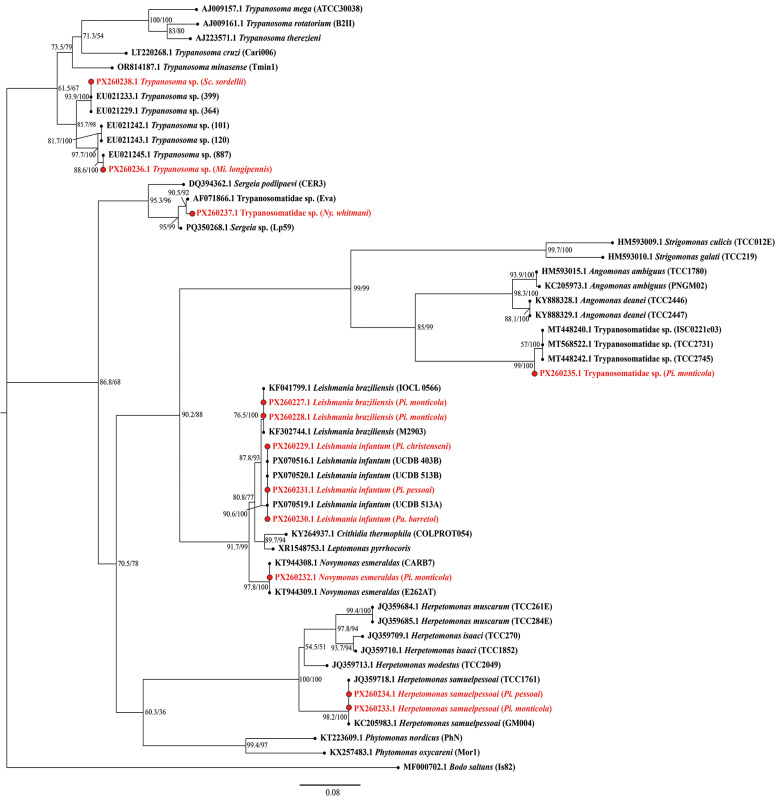
maximum likelihood (ML) phylogenetic tree of trypanosomatids based on the *18S rRNA* gene (V7-V8 region). Node support values are shown as SH-aLRT (%) / ultrafast bootstrap (UFBoot) replicates (%), based on 1,000 iterations. The tree was rooted with *Bodo saltans* (MF000702.1). Sequences generated in this study are highlighted in red.

**Fig. 4:**
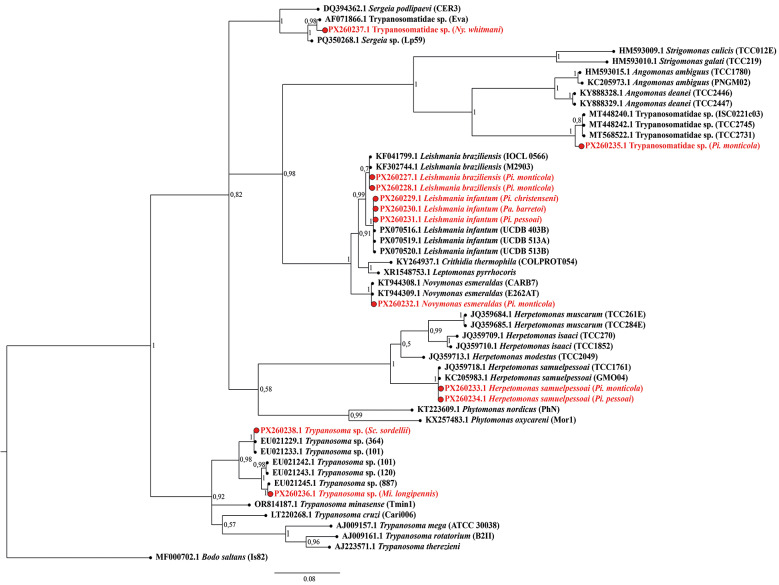
Bayesian inference (BI) phylogenetic tree of trypanosomatids based on the *18S rRNA* gene (V7-V8 region). Two independent runs of four chains each were performed for 5,000,000 generations, sampling every 100 generations and discarding the first 25% as burn-in. Posterior probabilities are indicated at nodes. The tree was rooted with *Bodo saltans* (MF000702.1). Sequences generated in this study are highlighted in red.

**Fig. 5:**
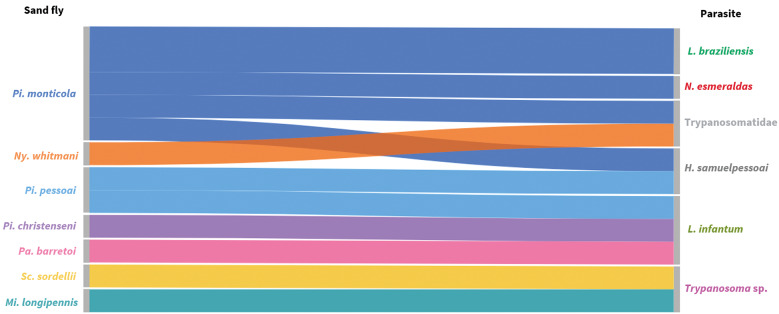
bipartite interaction diagram (Sankey diagram) between sand fly species and Trypanosomatidae identified during the study in the Serra do Cipó, Minas Gerais, Brazil.

For all positive samples, ML and Bayesian inference analyses yielded congruent topologies (Figs 3-4, respectively). The interaction matrix between the seven sand fly species that tested positive and the trypanosomatids detected in the study area reveals a complex network ranging from dixenous pathogens of medical relevance, such as *Leishmania* parasites, to less explored lineages, including *Novymonas*, *Trypanosoma* sp. from the Anura clade, and parasites related to *Sergeia* genus. Altogether, at least five subfamilies within Trypanosomatidae were represented (Fig. 5). Amplification of the mitochondrial *COI* gene was successful for all samples from both sylvatic and peridomestic settings, confirming DNA integrity and serving as an endogenous control [Supplementary data (Fig. 3)].

## DISCUSSION

Sand flies are widely recognised as vectors of *Leishmania*, but our results demonstrate that they can also harbour a broader spectrum of trypanosomatids, including poorly explored lineages. In the MT, we detected both *L. infantum* and *L. braziliensis*, as well as non-*Leishmania* taxa such as *H. samuelpessoai*, *N. esmeraldas*, a representative of Strigomonadinae, and *Trypanosoma* sp. from the Anura clade. In peridomestic settings, we additionally identified a lineage related to the genus *Sergeia* and another *Trypanosoma* sp. from the Anura clade. We also recorded *Sc. birali*, representing the first occurrence of this recently described species in Minas Gerais.^34^ Collectively, these findings reveal complex parasite-insect associations across sylvatic and peridomestic environments and reinforce the need to study sand flies beyond the narrow context of *Leishmania* ecology.

The species accumulation curve in the MT reached asymptote, and the observed richness (21 species) closely matched the *Chao 1* estimator, indicating that the sampling effort was sufficient to characterise the local community. Comparable surveys in nearby municipalities, such as Baldim,^35^ and Jaboticatubas,^36^ reported similar faunistic patterns, reinforcing that the richness observed in our study falls within the expected range for this region of Minas Gerais.

Species of *Pintomyia* (*Pintomyia*), particularly *Pi. fischeri* and *Pi. pessoai*, have been implicated as vectors of *L. infantum* and *L. braziliensis*, respectively,^18^ ^,^ ^37^ ^,^ ^38^ ^,^ ^39^ ^,^ ^40^ and are known to feed on humans and domestic animals.^41^ ^,^ ^42^ In MT, *Pi. pessoai* and *Pi. christenseni* were found carrying *L. infantum* DNA, suggesting their possible participation in wild-synanthropic transmission cycles of this parasite. Conversely, the role of species within *Pintomyia* (*Pifanomyia*) in *Leishmania* transmission remains debated. *Pintomyia monticola*, probably the most plausible putative vector of this subgenus, has been reported carrying DNA of both *L. infantum* and *L. braziliensis* in southeastern Brazil.^43^ ^,^ ^44^ In MT, *Pi. monticola* was positive for *L. braziliensis* DNA, and notably, it was observed feeding on humans during the day (9-11 a.m.). Such anthropophilic behaviour has been described previously.^45^ ^,^ ^46^ ^,^ ^47^ Vector competence studies are required to confirm whether this species can sustain late-stage infections of *Leishmania*, thus validating its vectorial role.

Another species of interest, *Pa. barretoi*, also tested positive for *L. infantum*. Ecological information on this species is scarce, as is the case for other members of *Psathyromyia* (*Forattiniella*). Among them, only *Pa. aragaoi* has been reported feeding on humans^48^ ^,^ ^49^ and testing PCR-positive for *L. braziliensis*.^50^ ^,^ ^51^ Our findings suggest that *Pa. barretoi* may have fed on hosts susceptible to *L. infantum* infection and could participate in wild or synanthropic transmission cycles of this parasite in MT. This reinforces that non-traditional sand fly species can also harbour *Leishmania* DNA, although the epidemiological significance of such detections remains uncertain.

Strikingly, the identification of non-*Leishmania* trypanosomatids broadens the spectrum of parasite diversity associated with sand flies. Reports of *Herpetomonas* and *Crithidia* in Brazilian sand flies have increased over the past decade,^8^ ^,^ ^9^ while the occurrence of *Novymonas* is, to our knowledge, described here for the first time in association with a sand fly. Although *Herpetomonas* is generally considered of limited epidemiological relevance, it has been reported infecting Egyptian rats^52^ and even in immunodepressed humans,^53^ fuelling the debate on the true extent of their monoxenous origin. In contrast, *N. esmeraldas*, the only species currently recognised in the genus, was originally described in the hemipteran *Niesthrea vincentii* (Hemiptera: Rhopalidae) from Ecuador.^54^ Phylogenetic and molecular evidence place *Novymonas* within the subfamily Leishmaniinae,^55^ providing valuable insights into the evolutionary origins of dixenous parasites within this lineage.^56^ In our study, BLAST analysis and both ML and Bayesian phylogenetic inferences consistently positioned the parasite detected in *Pi. monticola* as *N. esmeraldas*, supporting this taxonomic assignment. Nonetheless, given the substantial geographic distance between the type locality in Ecuador and our study area in Brazil, further molecular analyses using additional markers such as the full length of *18S rDNA*, *GAPDH* and *SL-RNA* genes would be necessary to confirm species identity. Such analyses could not be performed here due to limited DNA availability.

*Pintomyia monticola* was also found positive for a trypanosomatid identified as belonging to the subfamily Strigomonadinae.^57^ ^,^ ^58^ This group currently comprises the genera *Angomonas*, *Kentomonas*, and *Strigomonas*. Parasites of this lineage have been described in the Malpighian tubules, haemolymph, hemocoel, and particularly in the midgut of dipterans, which is considered the preferential site for multiplication and colonisation.^59^ *Strigomonas culicis*, for example, can colonise the insect midgut, subsequently invade the hemocoel, and eventually reach the salivary glands.^59^ ^,^ ^60^ In sand flies, a representative of *S. galati* has previously been reported in *Lu. almerioi* from Mato Grosso do Sul, Brazil.^57^ Interestingly, the parasite identified in *Pi. monticola* in our study was positioned in both ML and Bayesian inferences among sequences labelled as “Trypanosomatidae sp.” obtained from *Musca domestica* (Diptera: Muscidae) in Brazil (GenBank: MT448242.1 and MT568522.1) and from an unidentified dipteran in Barbados (MT448240.1). This cluster appears to be phylogenetically close to *Angomonas*, but due to the uncertain taxonomic resolution, we reported our detection as “Trypanosomatidae sp” like those sequences previously deposited.

A parasite closely related to the genus *Sergeia* (here referred to as “Trypanosomatidae sp”; GenBank PX260237) was detected in a female of *Ny. whitmani* from a peridomestic site. Species of *Sergeia*, such as *S. podlipaevi*, have been reported in the gut and Malpighian tubules of two biting midge species, *Culicoides* (*Oecacta*) *festivipennis* and *C.* (*Oecacta*) *truncorum*. Experimental infections in *C.* (*Monoculicoides*) *nubeculosus* demonstrated that by the fifth day post-infection, flagellates complete their development in the gut and most localise to the Malpighian tubules.^61^ However, *Sergeia* parasites are probably not restricted to biting midges, as phylogenetic analyses revealed an affinity between *S. podlipaevi* and Trypanosomatidae sp “strain EVA”, isolated in Venezuela from *Lu. evansi.* ^61^ Altogether, these results indicate that *Sergeia* parasites may not be restricted to biting midges but instead occupy a broader ecological niche that includes sand flies, raising questions about the evolutionary pathways and host associations of these trypanosomatids.

The detection of *Mi. longipennis* and *Sc. sordellii* carrying *Trypanosoma* sp DNA, previously described in anurans,^62^ corroborates the feeding habits reported for species of these genera, which are frequently associated with cold-blooded hosts, despite occasional records of feeding on warm-blooded animals.^63^ ^,^ ^64^ Several studies have documented the occurrence of *Trypanosoma* ^65^ ^,^ ^66^ ^,^ ^67^ and *Leishmania* ^51^ ^,^ ^68^ ^,^ ^69^ in *Micropygomyia* species, suggesting that they are susceptible to trypanosomatid infections. For *Trypanosoma*, the evidence clearly indicates that both *Micropygomyia* and *Sciopemyia* can host these parasites. In contrast, for *Leishmania*, the evidence remains insufficient to establish a vectorial role, which is still a matter of debate.

From an ecological perspective, the Serra do Cipó district, where MT is located, represents a landscape in which sylvatic boundaries are blurred by human settlement, forest fragmentation, and the overlap of domestic and wild reservoirs. Such contexts not only sustain the persistence of *Leishmania* transmission but may also facilitate incidental associations with other trypanosomatids. Species such as *Lu. longipalpis*, *Ny. whitmani*, and *Ev. cortelezzii* are well known for their ability to exploit anthropised habitats, and their presence in peridomestic areas reinforces their role in maintaining leishmaniasis cycles.^5^ ^,^ ^16^ ^,^ ^70^ The detection of non-*Leishmania* trypanosomatids further suggests that sand flies may act as ecological “hubs”, transiently interacting with parasites whose life cycles remain poorly understood. Whether these associations represent accidental infections, dead-end interactions, or potential adaptive processes remains an open question. In conclusion, our findings demonstrate that although the medical relevance of most of these parasites remains uncertain, the hidden diversity of trypanosomatids highlights the need to look beyond *Leishmania* in sand fly research, adopting a broader ecological perspective.

## SUPPLEMENTARY MATERIALS

Supplementary material

## Data Availability

All relevant data supporting the findings of this study are included within the manuscript and its Supplementary data. DNA sequences generated in this study have been deposited in GenBank under accession numbers PX260227 - PX260238. Additional datasets generated and/or analysed during the current study are available from the corresponding author upon reasonable request.
