# Supplementary material for: Beyond Leishmania: hidden trypanosomatid diversity reveals complex parasite-sand fly networks in southeastern Brazil
Source: Mem Inst Oswaldo Cruz. 2026 May 1;121:e250260. doi: 10.1590/0074-02760250260 (PMC13143167; doi:10.1590/0074-02760250260)
Supplement: Supplementary material [file 1678-8060-mioc-121-e250260-s1.pdf]

TABLE I

Sand fly collection sites at Serra do Cipó district (Mata da Tapera Municipal Natural Park - MT and peridomestic settings), Minas Gerais, Brazil

Site	Lat	Long	Ecotope
MT01	19°19’48.18”S	43°36’56.89”W	Sylvatic area
MT02	19°19’49.02”S	43°36’58.52”W	Sylvatic area
MT03	19°19’49.83”S	43°36’59.65”W	Sylvatic area
MT04	19°19’50.87”S	43°36’59.30”W	Sylvatic area
MT05	19°19’51.74”S	43°37’0.52”W	Sylvatic area
MT06	19°19’51.52”S	43°36’54.71”W	Sylvatic area
MT07	19°19’51.87”S	43°36’53.76”W	Sylvatic area
MT08	19°19’52.20”S	43°36’53.08”W	Sylvatic area
MT09	19°19’52.56”S	43°36’52.35”W	Sylvatic area
MT10	19°19’52.84”S	43°36’51.58”W	Sylvatic area
MT11	19°19’57.36”S	43°36’54.46”W	Sylvatic area
MT12	19°19’57.78”S	43°36’52.74”W	Sylvatic area
MT13	19°19’58.60”S	43°36’51.60”W	Sylvatic area
MT14	19°19’59.29”S	43°36’50.64”W	Sylvatic area
MT15	19°19’58.83”S	43°36’53.91”W	Sylvatic area
MT16	19°19’56.14”S	43°36’59.67”W	Sylvatic area
MT17	19°19’57.55”S	43°36’57.83”W	Sylvatic area
MT18	19°19’59.78”S	43°36’56.02”W	Sylvatic area
MT19	19°20’0.03”S	43°36’54.47”W	Sylvatic area
MT20	19°20’0.89”S	43°36’57.73”W	Sylvatic area
P1	19°20’3.32”S	43°37’0.85”W	Peridomestic area
P2	19°20’5.47”S	43°36’58.97”W	Peridomestic area
P3	19°20’10.08”S	43°36’57.70”W	Peridomestic area
P4	19°20’11.41”S	43°36’57.25”W	Peridomestic area
P5	19°20’11.87”S	43°36’57.14”W	Peridomestic area
P6	19°19’48.32”S	43°37’2.23”W	Peridomestic area
P7	19°19’43.32”S	43°36’55.91”W	Peridomestic area
P8	19°19’49.27”S	43°36’54.54”W	Peridomestic area
P9	19°19’47.77”S	43°36’52.05”W	Peridomestic area

TABLE II

Sand flies collected with CDC light traps and Shannon trap, by sex, during 2023 and 2024 in the Mata da Tapera (MT), Serra do Cipó district, Minas Gerais, Brazil

	Mar 23		Jun 23		Sep 23		Jan 24		Jul 24		
Sand fly	♂	♀	♂	♀	♂	♀	♂	♀	♂	♀	Total (%)
Brumptomyia brumpti	2	1	1	2	0	0	3	2	0	0	11 (0.9)
Evandromyia bacula	0	0	0	1	0	1	0	1	0	0	3 (0.2)
Evandromyia cortelezzii	1	0	2	2	0	4	5	7	3	4	28 (2.3)
Evandromyia evandroi	8	5	1	5	2	4	7	8	2	1	43 (3.5)
Evandromyia teratodes	0	2	0	0	0	0	0	0	0	0	2 (0.2)
Evandromyia termitophila	0	0	0	0	0	1	1	0	0	0	2 (0.2)
Lutzomyia ischnacantha	0	5	1	2	0	0	0	0	0	0	8 (0.7)
Lutzomyia longipalpis	12	1	6	0	0	0	3	3	5	0	30 (2.4)
Micropygomyia longipennis	0	1	0	0	0	0	0	3	2	0	6 (0.5)
Micropygomyia quinquefer	0	1	0	0	0	0	0	0	0	4	5 (0.4)
Migonemyia migonei	0	0	0	0	2	0	0	0	0	0	2 (0.2)
Nyssomyia whitmani	3	3	3	3	5	10	5	2	12	4	50 (4.1)
Pintomyia christenseni	2	0	3	3	12	18	12	13	0	18	81 (6.6)
Pintomyia monticola	3	24	9	22	22	78	17	73	37	53	338 (27.6)
Pintomyia pessoai	20	10	7	10	102	111	44	31	121	38	494 (40.3)
Psathyromyia aragaoi	3	1	20	4	0	0	18	15	5	2	68 (5.5)
Psathyromyia barretoi	0	2	0	21	0	0	0	0	0	0	23 (1.9)
Psathyromyia brasiliensis	0	1	0	0	0	0	0	2	0	0	3 (0.2)
Psathyromyia lutziana	0	3	3	1	0	0	0	1	0	1	9 (0.7)
Sciopemyia birali	0	0	0	0	0	0	0	1	0	0	1 (0.1)
Sciopemyia sordellii	4	2	0	5	1	1	1	3	0	2	19 (1.5)
Total	58	62	56	81	146	228	116	165	187	127	1.226 (100)
	120 (9.7)		137 (11.1)		374 (30.5)		281 (22.9)		314 (25.8)		

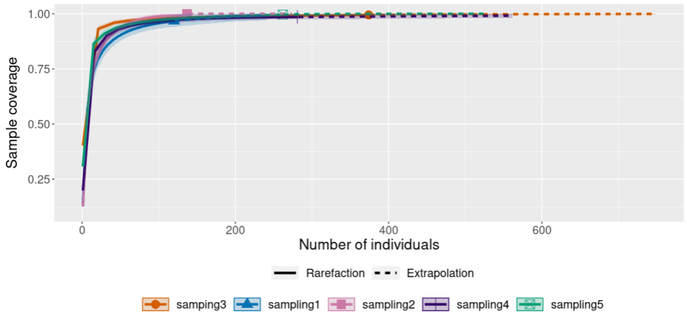

Fig. 1: sample completeness curves for sand fly collections in Mata da Tapera Municipal Natural Park (MT). Sample coverage (solid lines) with extrapolation (dashed lines) was estimated using the iNEXT framework based on abundance data, with 200 bootstrap replications to compute 95% confidence intervals (shaded areas). All curves reached values above 95% coverage, indicating that the sampling effort was sufficient to capture the great majority of species present in the study area.

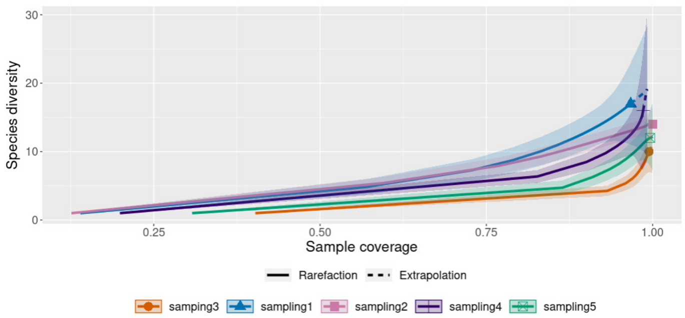

Fig. 2: coverage-based rarefaction and extrapolation of sand fly species richness. Coverage-based rarefaction (solid lines) and extrapolation (dashed lines) curves were generated using the iNEXT framework with 200 bootstrap replications to compute 95% confidence intervals (shaded areas). The x-axis represents sample coverage (completeness of sampling), and the y-axis represents observed and estimated species richness (q = 0). All curves converged to high coverage values (> 95%), indicating that the sampling effort was sufficient to capture most of the sand fly diversity in the study area.

TABLE III

Sand flies collected with CDC light traps in January 2025 in peridomestic settings surrounding the Mata da Tapera (MT), Serra do Cipó district, Minas Gerais, Brazil

	Collection sites																		
	P1		P2		P3		P4		P5		P6		P7		P8		P9		
Sand fly	♂	♀	♂	♀	♂	♀	♂	♀	♂	♀	♂	♀	♂	♀	♂	♀	♂	♀	Total (%)
Br. brumpti	0	1	0	0	0	0	1	0	0	0	0	0	0	0	0	0	0	0	2 (0.9)
Ev. cortelezzii	12	17	1	0	0	0	1	0	1	0	1	1	7	0	0	0	0	0	41 (17.5)
Ev. evandroi	2	0	1	1	0	0	0	0	1	0	1	0	32	29	2	2	0	0	71 (30.3)
Lu. longipalpis	3	0	0	0	0	0	0	0	0	0	1	2	50	3	0	0	0	0	59 (25.2)
Mi. migonei	0	0	0	0	0	0	0	0	0	0	0	1	3	0	0	0	0	0	4 (1.7)
Ny. whitmani	0	1	0	0	1	0	0	0	0	1	0	0	7	3	0	0	0	1	14 (6.0)
Pi. christenseni	0	6	0	0	0	0	0	0	0	0	0	0	0	0	0	0	0	1	7 (3.0)
Pi. monticola	2	0	1	0	0	0	0	0	0	0	0	0	0	1	1	0	0	1	6 (2.6)
Pi. pessoai	0	0	0	0	0	1	0	0	0	0	2	0	17	4	0	0	0	0	24 (10.3)
Sc. birali	0	1	0	0	0	0	0	0	0	0	0	0	0	0	0	0	0	0	1 (0.4)
Sc. sordellii	0	2	0	1	0	0	0	1	0	0	0	0	0	1	0	0	0	0	5 (2.1)
Total (%)	19	28	3	2	1	1	2	1	2	1	5	4	116	41	3	2	0	3	234 (100)
	47 (20.0)		5 (2.1)		2 (0.8)		3 (1.3)		3 (1.3)		9 (3.8)		157 (67.3)		5 (2.1)		3 (1.3)		

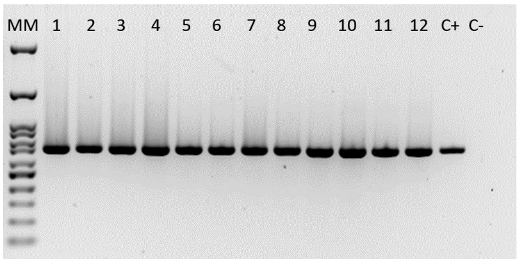

Fig. 3: representative agarose gel electrophoresis (2%) of genomic DNA integrity through the amplification of *COI* gene, used as endogenous controls to confirm the quality and integrity of the extracted genomic material. MM: molecular weight marker (100 bp ladder); Lanes 1-12: *COI* gene amplification products from sand flies; C+: positive control (*Lutzomyia longipalpis* DNA); C-: negative control (PCR mix without DNA template).
